# Quantification of carotid plaque lipid content with magnetic resonance T2 mapping in patients undergoing carotid endarterectomy

**DOI:** 10.1371/journal.pone.0181668

**Published:** 2017-07-26

**Authors:** Mohammad Alkhalil, Luca Biasiolli, Joshua T. Chai, Francesca Galassi, Linqing Li, Christopher Darby, Alison Halliday, Linda Hands, Timothy Magee, Jeremy Perkins, Ed Sideso, Peter Jezzard, Matthew D. Robson, Ashok Handa, Robin P. Choudhury

**Affiliations:** 1 Acute Vascular Imaging Centre, Radcliffe Department of Medicine, University of Oxford, Oxford, United Kingdom; 2 FMRIB Centre, Nuffield Department of Clinical Neurosciences, University of Oxford, Oxford, United Kingdom; 3 Nuffield Department of Surgical Sciences, University of Oxford, Oxford, United Kingdom; Heart Research Institute, AUSTRALIA

## Abstract

**Background and purpose:**

Techniques to stratify subgroups of patients with asymptomatic carotid artery disease are urgently needed to guide decisions on optimal treatment. Reliance on estimates of % luminal stenosis has not been effective, perhaps because that approach entirely disregards potentially important information on the pathological process in the *wall* of the artery.

**Methods:**

Since plaque lipid is a key determinant of plaque behaviour we used a newly validated, high-sensitivity T2-mapping MR technique for a systematic survey of the quantity and distribution of plaque lipid in patients undergoing endarterectomy. Lipid percentage was quantified in 50 carotid endarterectomy patients. Lipid distribution was tested, using two imaging indices (contribution of the largest lipid deposit towards total lipid (LLD %) and a newly-developed LAI ‘lipid aggregation index’).

**Results:**

The bifurcation contained maximal lipid volume. Lipid percentage was higher in symptomatic *vs*. asymptomatic patients with degree of stenosis (DS ≥ 50%) and in the total cohort (P = 0.013 and P = 0.005, respectively). Both LLD % and LAI was higher in symptomatic patients (P = 0.028 and P = 0.018, respectively), suggesting that for a given plaque lipid volume, coalesced deposits were more likely to be associated with symptomatic events. There was no correlation between plaque volume or lipid content and degree of luminal stenosis measured on ultrasound duplex (r = -0.09, P = 0.53 and r = -0.05, P = 0.75), respectively. However, there was a strong correlation in lipid between left and right carotid arteries (r = 0.5, P <0.0001, respectively).

**Conclusions:**

Plaque lipid content and distribution is associated with symptomatic status of the carotid plaque. Importantly, plaque lipid content was not related to the degree of luminal stenosis assessed by ultrasound. Determination of plaque lipid content may prove useful for stratification of asymptomatic patients, including selection of optimal invasive treatments.

## Introduction

The European Carotid Surgery Trial (ESCT) and the North American Symptomatic Carotid Endarterectomy Trial (NASCET) have demonstrated the efficacy of carotid endarterectomy (CEA) in reducing risk of ischaemic stroke in patients with recent symptomatic carotid artery disease.[1,2] Luminal stenosis and time of surgery were identified as important determinants of benefits from CEA in the symptomatic patients.[3] On the other hand, the benefit of CEA in the asymptomatic patients has been more controversial.[4] The Asymptomatic Carotid Artery Study (ACAS) and the Asymptomatic Carotid Surgery Trial (ACST) have demonstrated a potential role of CEA mainly in patients with significant % luminal stenosis (> 60–70%).[5,6] Notably, CEA was highly favourable in the symptomatic patients with an absolute risk reduction of 16% compared to a modest 5–6% in the asymptomatic ones with similar degree of stenosis.[5–7] Moreover, medical therapy has significantly evolved and more patients are currently on antiplatelet, antihypertensive and lipid-lowering drugs.[8] This has led into significant reduction in annual risk of stroke within the ACST cohort, from 2.4% annually to 1.4%.[9] This is particularly important given that 30-day risk of stroke after CEA in the CREST (Stenting versus Endarterectomy for Treatment of Carotid Artery Stenosis) trial was 2.3%.[10] Therefore, there is an urgent need to identify a subgroup of asymptomatic patients who potentially may benefit from revascularisation and as such it would not be offset by the risk of the procedure itself.[4]

Given the lack of uniformity of stroke risk in patients with asymptomatic carotid artery disease, clinical and imaging tools have been applied in an attempt to identify asymptomatic carotid patients with high risk of stroke.[4] Importantly the % luminal stenosis, estimated from ultrasound duplex, has not been shown to be useful to stratify risk in asymptomatic patients.[4] Both ACAS and ACST trials showed no relationship between % luminal stenosis and future risk of stroke in asymptomatic carotid patients.[5,6] This should not be surprising since % luminal stenosis almost entirely disregards the pathological atherosclerotic lesion, which resides in the wall of the artery with highly variable incursion to the lumen. On the other hand, plaque characteristics such as large lipid content is associated with advanced atherosclerosis and with high risk of plaque rupture leading to acute vascular events.[11,12] In large patient cohorts, estimation of increased lipid core on T1-weighted images of carotid MRI predicted future cardiovascular risks.[13,14] Similarly, it is plausible that homogenous fibrotic carotid plaques carry lower risk and aggressive medical therapy may be optimal for these patients.

We have recently developed and validated an MRI technique (T2 mapping) that accurately and objectively quantifies carotid plaque lipid on a voxel by voxel basis, without reliance on the presence of discernible lipid core.[15,16] Accordingly, we sought to study whether plaque lipid *content* and *distribution* can distinguish symptomatic from asymptomatic plaques, and potentially identify those who are more likely to become symptomatic. In addition, we studied if there is any relationship between plaque lipid and % lumen stenosis and whether some patients have propensity to develop multi-site lipid-rich plaques as potential indication for more aggressive medical therapy, such as monoclonal antibodies targeting PCSK9. Matching specific targeted therapy (lipid-lowering effect) with disease characteristics (e.g. lipid accumulation at multiple vascular sites) could provide opportunities to refine the use of these new and expensive anti-atherosclerotic drugs.[17]

## Materials and methods

### Study population

71 patients scheduled for carotid endarterectomy at Oxford University Hospitals NHS Trust were recruited from November 2011 to September 2015. Patients were scanned at the Oxford Acute Vascular Imaging Centre (AVIC) ≤ 24 hours before surgery. Patients were adult (> 18 years), had recently symptomatic (median time from index event 2 weeks) or asymptomatic carotid disease, with 50–99% carotid stenosis according to NASCET, or 70–99% according to ESCT criteria.[15,18] Plaques were defined as ‘culprit’ plaques where they were deemed to have given rise to either a minor cerebrovascular accident (CVA) or a transient ischaemic attack (TIA) as assessed clinically and supported, where available, by brain MRI / computed tomography imaging. Asymptomatic carotid plaques were those that had no documented clinical symptoms, but with an indication for carotid endarterectomy based on degree of stenosis. Ethical approval was obtained from National Research Ethics Services (NRES) and local R&D committee prior to commencement of the study and all participants provided written informed consent. Some (n = 24) of these patients were included in the histological validation of T2 mapping reported by Chai *et al*.[15]

### MRI protocol

Patients were imaged on a Verio 3T scanner (Siemens Healthcare, Erlangen, Germany) with a 4-channel phased-array carotid coil (Machnet, Roden, The Netherlands). Bright-blood time-of-flight (TOF) angiography of the carotid arteries was acquired to localize carotid bifurcation and atherosclerotic plaque. Multi-slice carotid T_2_ maps were generated from 14 images with echo times TE = 9–127 ms and repetition time TR = 2 s, acquired using the DANTE-MESE sequence that combined black-blood preparation based on non-selective Delay Alternating with Nutation for Tailored Excitation (DANTE) pulse trains[19,20] with chemical-shift-selective fat saturated Multiecho Spin-Echo (MESE). DANTE-MESE acquired 10 slices of 2 mm thickness each, covering 2 cm of the target carotid artery in 8 minutes. Slices were centralised on the maximum plaque volume identified on TOF and T1 weighted images so full plaque length was covered within 10 slices.

### Data analysis

T_2_ maps of the carotid arteries were generated *voxel-by-voxel* using mono-exponential nonlinear fitting,[16] and lumen and external vessel boundaries were segmented using a validated semi-automated procedure.[21] A segmentation method to identify lipid was implemented using T2 threshold (<42 ms) as previously validated.[15] Each vessel (right and left) had 10 slices studied covering 2 cm of carotid territory (5 slices were obtained in 7 patients). All algorithms were implemented in Matlab (MathWorks, Natick, USA).

Plaque lipid and plaque volume were quantified using a single voxel as one unit (0.33 x 0.33 x 2 mm). Voxels with T2 values of less than 42 ms threshold range were grouped together to quantify lipid volume (without recent intra-plaque haemorrhage), according to the published method.[15] Lipid and plaque volumes of the studied vessel were calculated using the average lipid and plaque volume in all the 10 studied slices respectively and irrespective of the degree of stenosis (unit of analysis).

Lipid distribution was studied based on percentage and number of separate lipid deposits within the slice. We identified slices with the maximum lipid volume on both sides (right and left) and subsequently quantified number of separate lipid deposits in that slice. We then calculated the percentage of every individual lipid deposit out of the total lipid area of the studied slice. We aimed to test whether there were important differences not only in lipid content but also in lipid distribution, in terms of scattered lipid deposits *vs*. coalesced ‘pools’. To achieve this, we applied two imaging indices in which we identified the largest lipid deposit (LLD) within the selected slice (slice with the largest lipid volume). We then calculated its contribution (percentage LLD %) to the total slice lipid volume and this represented the first tested imaging index.

Subsequently, we averaged the remainder of lipid deposits percentage to assess if these deposits add any insights towards the symptomatic status of the plaque (RLD %) (excluding the largest one): RLD% = 100 * (1-LDD) / (number of lipid deposits-1) *(For number of lipid deposits ≥ 2)*. Lipid aggregation index (LAI) is the ratio of largest lipid deposit percentage (LLD %) and other lipid deposits (RLD %): *LAI =* LLD% / RLD% (S1 Fig). Accordingly, a plaque with a large deposit would have a high LAI; whereas a plaque with multiple small deposits would have a lower LAI, even if the total lipid content were similar. LAI was not applicable in patients with single lipid deposit as (a) there is no merit of quantifying lipid scattering if all lipid deposits are coalesced in a single deposit and (b) the index will not be calculable. This process was executed by using ImagePro Plus software (Media Cybernetics, Rockville, USA), where lipid deposits of very small size (≤ 1%) were excluded to be distinguished from noise. Lipid area and number of lipid particles were segmented and quantified by an operator blinded to the origin of the data.

### Statistical analysis

Data were expressed as frequencies and percentages for categorical variables, mean and (±) standard deviation for continuous variables or as median accompanied by interquartile range (IQR) for skewed continuous variables, as appropriate. In detail, parameters of *plaque lipid contents* including lipid percentage, lipid volume, LAI, and LDD% were shown as median and IQR as the Shapiro-Wilk test showed the data were non-normally distributed.

Categorical baseline characteristics were compared using Chi squared (X^2^) test or Fisher’s Exact test (FET) where appropriate, while continuous baseline characteristics were compared using unpaired student t-test between symptomatic and asymptomatic patients (all normally distributed). Wilcoxon rank sum tests was used to test difference in lipid percentage and distribution as data were not normally distributed.

Correlations were assessed using Spearman rank test if one or both covariates violated assumptions of Pearson R coefficient.

Statistical analysis was performed using SPSS 22.0 (SPSS, Inc., Chicago, IL, USA) and *P*-values <0.05 were considered statistically significant.

## Results

A total of 71 patients were recruited during the study period. 21 patients were excluded due to inadequate MRI image quality (29.5%), mostly due to motion artefact. In the remaining 50 patients, 16 were asymptomatic and 34 had prior symptoms that were attributable to the downstream cerebrovascular territory of one or other carotid artery. The mean age ± SD was 70 ± 12 years with overall mean stenosis, measured by duplex ultrasound, of 80 ± 10%, which was not significantly different between the 2 groups. Baseline patient characteristics are summarized in Table 1 and categorized according to presence or absence of symptoms. There were no significant differences in age [71 (± 12) *vs*. 67 (± 13), unpaired t-test P = 0.25], gender [79% vs. 63%, Chi squared test P = 0.30 for male gender], major cardiovascular risk factors (Chi squared test P = NS) or medications (Chi squared test P = NS) on admission between symptomatic and asymptomatic groups.

**Table 1:** Baseline characteristics of recruited patients.

	Symptomatic	Asymptomatic	Statistical test, P-value
Total	34	16	
Male (%)	27 (79%)	10 (63%)	X^2^ test, 0.30
Mean age ± SD	71 (± 12)	67 (± 13)	Unpaired t-test, 0.25
**Cardiovascular risk factors**			
Hypertension	27 (79%)	10 (63%)	FET, 0.30
Hypercholesterolemia	20 (59%)	11 (69%)	X^2^ test, 0.50
Smoking	16 (47%)	7 (44%)	X^2^ test, 0.80
Diabetes mellitus	7 (21%)	4 (25%)	FET, 0.72
CAD disease	7 (21%)	2 (13%)	FET, 0.69
Atrial fibrillation	7 (21%)	0 (0%)	FET, 0.08
**Medication at time of carotid endarterectomy**			
Aspirin /antiplatelets	26 (76%)	12 (75%)	FET, 1.0
Statins	27 (79%)	13 (81%)	FET, 1.0
Beta-blockers	5 (15%)	4 (25%)	FET, 0.42
Calcium antagonists	9 (26%)	3 (23%)	FET, 0.70
ACE inhibitors ARBs	11 (32%)	8 (50%)	X^2^ test, 0.17
Anticoagulation	4 (12%)	0 (0%)	FET, 0.29
**Duplex ultrasound scan**			
Right: Left	2.7: 1	1: 1	X^2^ test, 0.10
Mean stenosis ± SD (%)	78 ± 11%	83 ± 8%	Unpaired t-test, 0.08

SD, standard deviation; CAD, coronary artery disease; ARB, angiotensin receptor blockers, X^2^ Chi squared, FET Fisher exact test.

Plaque lipid was present in (8.99 ± 1.92) slices per vessel in the 100 obtained carotid vessels. The carotid bifurcation contained the maximal lipid content, irrespective of the symptomatic status or degree of stenosis, in 74.6% of the vessels (56 out of 75) where the bifurcation was included in the studied vessel.

**Fig 1:**
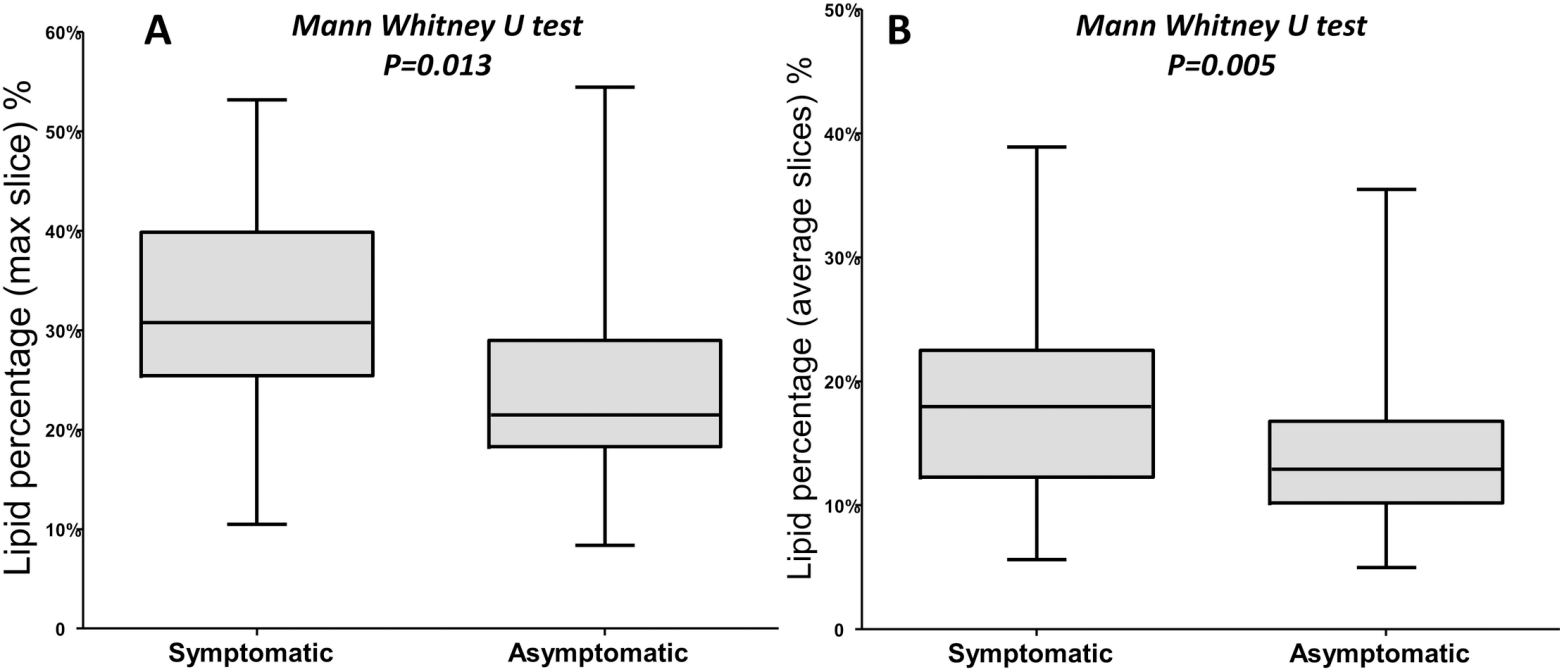
Difference in lipid percentage between symptomatic and asymptomatic patients. (A) Using slice with maximal lipid content, symptomatic patients are more likely to have lipid in patients with degree of stenosis of more than 50%. (B) Similarly but using averaged 10 slices, symptomatic patients have larger lipid percentage than asymptomatic ones in the total 100 carotid arteries.

Using the slice with maximal lipid in the carotid arteries with luminal stenosis ≥ 50%, symptomatic patients had larger lipid percentage than asymptomatic ones [30.8% (IQR 25.45–39.9) % *vs*. 21.5% (IQR 18.3–29) %, Mann-Whitney U test P = 0.013] (Fig 1A). Similarly, lipid percentage, calculated as average from all slices, was higher in the symptomatic vessel compared to the asymptomatic vessels within the total cohort (100 carotid vessels) [18% (IQR 12.3–22.5) % *vs*. 13% (IQR 10.2–16.8) %, Mann-Whitney U test P = 0.005] (Fig 1B).

**Fig 2:**
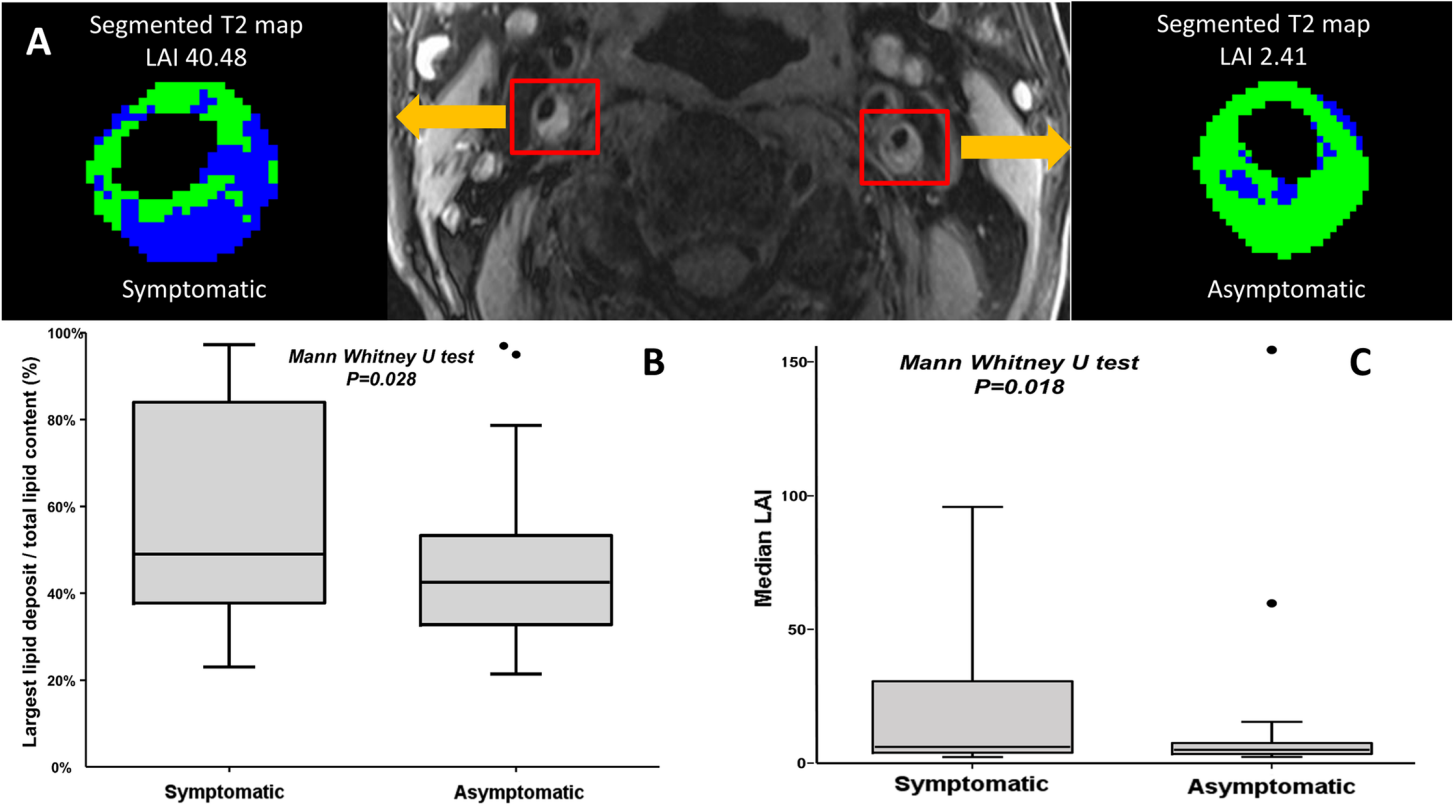
Assessment of lipid distribution using T2 mapping technique. (A) Cross sectional T1-weighted image of bilateral carotid arteries stenoses in the same patient. Segmented T2 map of the symptomatic right vessel of two weeks old history of transient ischaemic event (left sided facial weakness)- showing LAI of 40.48 suggestive of coalesced lipid compared to the left asymptomatic side with LAI of 2.41 (panel A). (B) Median LDD % is statistically higher in symptomatic patients compared to asymptomatic ones. (C) Similarly, median lipid aggregation index is statistically higher in symptomatic patients compared to asymptomatic ones. Notably two outlier vessels were identified with extremely high LDD % and LAI at the time of MRI where the corresponding plaque was yet still silent.

We defined two imaging indices namely ratio of largest lipid deposit over total lipid content (LDD %) and lipid aggregation index (LAI), where in both cases the higher the index, the more likely was a plaque to have a large individual lipid deposit (coalesced lipid pool) while plaques with low indices were more likely to contain scattered lipid deposits of more uniform size distribution (Fig 2A). The median LDD% was 45% (35–57%) while the LAI median was 5.11 (IQR = 3.68 to 8.81). Using these two indices we investigated the significance of patterns of lipid deposition in relation to the symptom-related versus non symptom-related status of plaques. Median LDD% was significantly higher in the symptomatic compared to the asymptomatic plaques [49% (IQR 38–84) % *vs*. 42% (IQR 32–53) %, Mann-Whitney U test P = 0.028] (Fig 2B). Similarly, median LAI was significantly higher in the symptomatic compared to the asymptomatic plaques [(6.16 (IQR 3.9–30.66) *vs*. 4.99 (IQR 3.47–7.56), Mann-Whitney U test P = 0.018] (Fig 2C), indicating that for a given quantity of plaque lipid, coalesced cores were more likely to be associated with symptomatic events.

**Fig 3:**
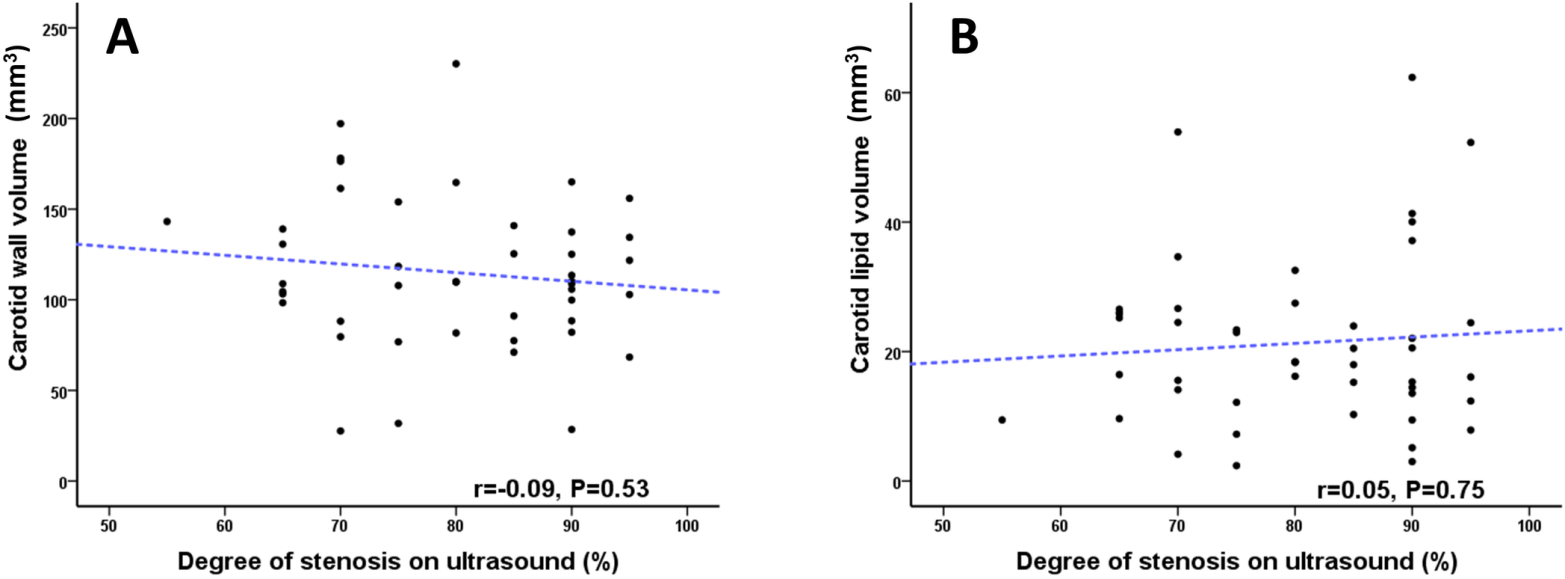
Plaque and lipid volume relationship with degree of luminal stenosis. (A) Scatter plot graph displaying the absence of correlation between degree of luminal stenosis measured on ultrasound duplex and carotid vessel wall volume. (B) Similarly scatter plot graph displaying the absence of correlation between degree of luminal stenosis measured on ultrasound duplex and carotid lipid volume.

There was no correlation between plaque volume and degree of luminal stenosis measured on ultrasound duplex (Spearman r = -0.09, P = 0.53) (Fig 3A). Nor was there any correlation between vessel lipid content and degree of stenosis (Spearman r = -0.05, P = 0.75) (Fig 3B).

**Fig 4:**
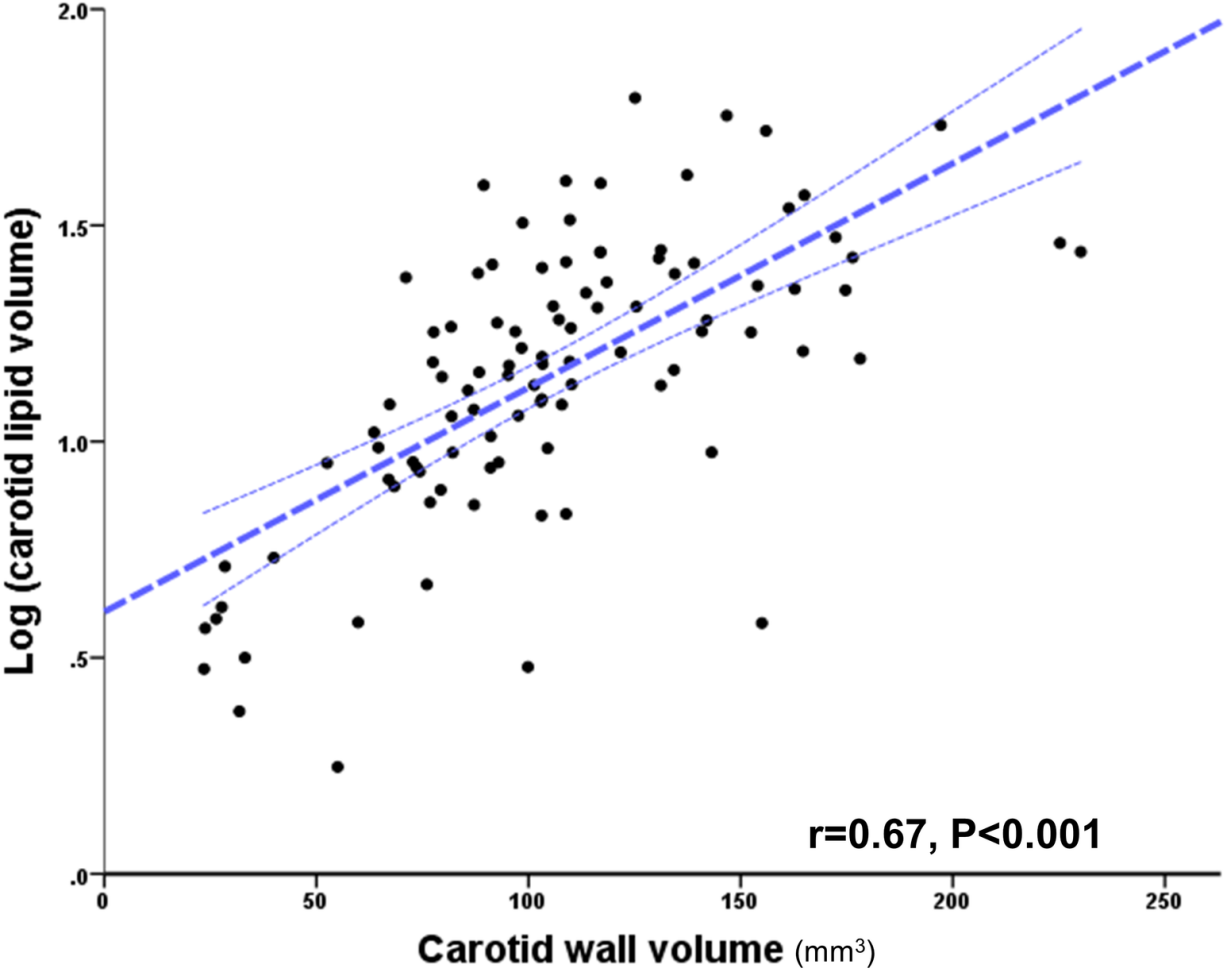
Correlation between carotid plaque and lipid volume (log transformed). Scatter plot graph illustrating a strong correlation between plaque lipid content and plaque volume in total of one hundred carotid arteries analysed using T2 map. Dotted lines indicate 95% confidence intervals. Note that increasing plaque size is associated with an increasing variation of lipid content; i.e. points fall further outside the 95% CI.

**Fig 5:**
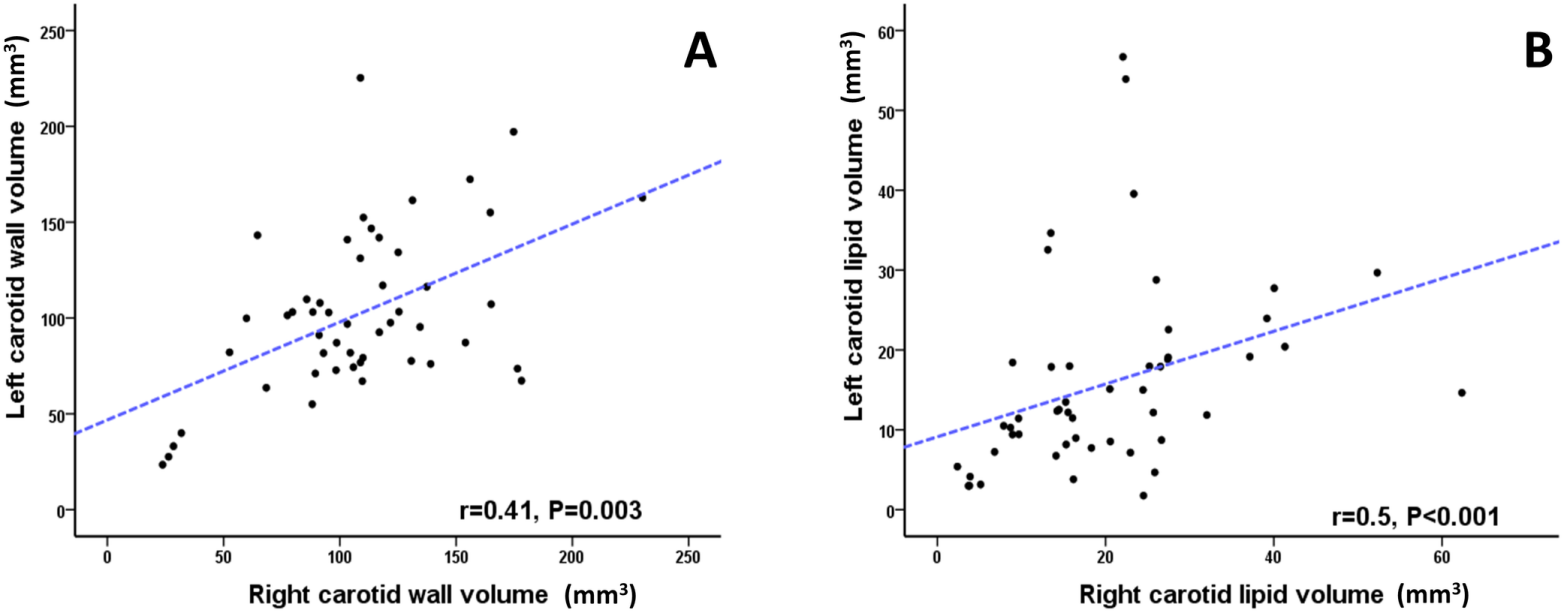
Spearman’s rank correlation between left and right carotid plaque and lipid volume. (A) Scatter plot graph demonstrating statistically significant correlation between plaque burden (total plaque volume) between left and right carotid arteries. (B) Scatter plot graph showing statistically significant correlation between lipid volumes of the right and left carotid arteries quantified on T2 map (panel B).

Comparing lipid and plaque volumes from the total 100 vessels of the cohort, and in order to evaluate variations with larger plaques using 95% confidence interval base 10 logarithmic transformation for lipid volume Log (*x*) was applied, there was a strong correlation between vessel lipid content and plaque volume (Pearson r = 0.67, P < 0.0001), but with larger plaques demonstrating larger variations in lipid content (Fig 4). In addition, there were statistically significant correlations between left and right carotid plaque volume (Spearman r = 0.41, P = 0.003) (Fig 5A) and absolute plaque lipid volume (Spearman r = 0.5, P <0.0001) (Fig 5B). Furthermore, there was no statistical significance between right and left carotid plaque volume (108 ± 41 mm^3^ *versus* 102 ± 42 mm^3^, P = 0.30 respectively), suggesting systemic response in plaque development.

## Discussion

Using a newly-validated *non-invasive* T2 mapping MRI technique we undertook a systematic quantification of carotid plaque lipid in patients undergoing carotid endarterectomy. The principal findings were that: (1) A large majority of plaques contained lipid, which tended to be maximal at the carotid bifurcation; (2) Plaque lipid *content* was higher in symptomatic versus asymptomatic arteries; (3) Plaque lipid *distribution* was related to the symptomatic status of the plaque, where more coalescence, as opposed to scattered lipid, was associated with prior symptoms; (4) % Luminal stenosis, measured by ultrasound duplex, did not reflect either atherosclerosis burden or lipid volume; (5) lipid content was strongly correlated between left and right carotid arteries.

Despite the ample evidence that plaque structure and composition are important determinants of both spontaneous and peri-procedural stroke risk,[22–24] current methods for determining which patients progress to surgical intervention rely not on assessment of the disease process in the wall of the artery, but on estimation of the % lumen stenosis. As the current study demonstrates, % lumen stenosis does not reflect burden of disease or lipid content. However, T2 mapping with MRI quantified plaque burden and lipid, which was higher in symptomatic *vs*. asymptomatic plaques.

Of patients with asymptomatic carotid stenosis with unilateral or bilateral % luminal stenosis of at least 60%, only a small minority (10.8%) have stroke at ten–year follow up.[9] Moreover, surgical intervention is associated with an appreciable risk.[10] Previous reports have stated the need for a tool that could identify both lower risk patients (who could pursue medical therapy) from higher risk group patient (potentially candidates for revascularisation).[4]

Similarly, carotid artery stenting is associated with significant risk of periprocedural stroke.[10] Interestingly, carotid plaque composition, in particular large plaque lipid volume, assessed using intra-vascular ultrasound was associated with large amount of debris during carotid stenting and is potentially an important factor in causing peri-procedural stroke.[24] These findings are consistent with outcomes in the CREST trial,[10] where there was no difference between stent and surgery in asymptomatic group compared to better outcomes of surgery in the symptomatic group.[10] Taken together these observations suggest that there may be a rationale to select patients for vascular intervention (stent vs. endarterectomy) based on pre-operative, ideally non-invasive, assessment of plaque volume and lipid burden.

Here we show that at the individual patient level of analysis, quantitative T2 mapping, showed differences related to the symptomatic status of carotid plaque, based on lipid volume. Our data extend previous observations comparing mean plaque lipid content averaged between multiple symptomatic versus asymptomatic vessels.[15] Moreover, we have demonstrated that in addition to lipid volume, the pattern of lipid distribution also reflects the symptomatic status of the plaque, where coalesced lipid is more likely to be associated with events. Two ‘outlier’ vessels were identified with extremely high LAI at the time of MRI, but in plaques had not [yet] declared symptoms. We hypothesize that prospective studies may show such plaques to be at particularly high future risk.

Larger plaques contained increasing quantities of lipid but, importantly, the quantity of plaque lipid in relation to plaque size became less predictable in large plaques, emphasizing the importance of individual plaque lipid quantification. Similar to previous reports using intra vascular ultrasound coupled with virtual histology (IVUS-VH) and near infra-red spectroscopy (NIRs) of the coronary arteries,[25] we showed using a non-invasive technique that the larger the plaque volume the greater is the likelihood of having a larger lipid volume. Thus lipid content may play a role in decision making with regards to carotid intervention.

Plaque lipid volume correlated between left and right carotid arteries, suggesting that some patients may have a propensity to develop more or less lipid-rich plaques. Atherosclerosis is a systematic process where atherosclerotic plaques are identified at multiple sites in some individuals,[26] and the ability to stratify patients based on the extent and nature of atherosclerotic plaques is a promising strategy for selecting patients for specific intensive treatments, akin to the imaging-based staging that is used to guide selection of cancer treatments.[17] Studies comparing atherosclerosis between left and right carotid arteries are sparse.[27,28] In 50 pairs of carotid arteries from cadavers, total wall volumes on MRI of left and right carotid arteries were correlated.[28] We confirmed these findings *in vivo* in a more clinically relevant cohort of high-risk atherosclerotic patients and extended the observation to include not only plaque size but composition. In the Rotterdam study the prevalence, severity and composition of left and right carotid arteries were assessed using non-contrast MRI in stroke-free population.[27] Bilateral atherosclerotic plaques were identified in most individuals (85%) reflecting the systemic nature of atherosclerosis. With emerging new lipid lowering drugs like PCSK9 inhibitors,[29,30] identifying a group of patients with lipid-rich plaques may offer a potential targeted indication for these drugs.

The main limitation of our method is the sensitivity to motion artefacts of the DANTE-MESE sequence, a fact reflected in our relatively high overall rejection rate (29.5%) of all scans. However, there has been marginal improvement in using this technique compared since our initial rejection rate of 35%.[15] In addition, we are currently developing motion correction strategies, so our technique will fulfil its full clinical potential. Another limitation is related to LAI where it is not calculable in slices with single lipid deposit and therefore it only should be applied when number of lipid deposits is ≥2. However, this is a theoretical limitation as there was no slice with a single lipid deposit within the total cohort.

In summary, lipid content and distribution is related to the plaque’s symptomatic status and the relationship between individual carotid arteries allow further refinement to identify those at high risk, beyond luminal stenosis. This highly-sensitive T2 mapping MRI technique could allow a more individualised approach to patient management, informing decisions on medical and surgical interventions and maybe of use in assessing response to treatment in clinical trials.

## Supporting information

S1 Fig Lipid aggregation index calculation. Cross-sectional slice of carotid artery demonstrating five different lipid deposits labelled alphabetically (a, b, c, d & e) and identified on T2 map technique. Lipid deposit (e) was excluded from the analysis as it counts ≤ 1%. LDD% is the percentage of deposit (a) out of the total lipid deposits (a/a + b + c + d) while RDD % = (1-a) or (b + c + d) divided by three in this example.
(TIF)
