# Supplementary figures and images for: Quantification of carotid plaque lipid content with magnetic resonance T2 mapping in patients undergoing carotid endarterectomy
Source: PLoS One. 2017 Jul 26;12(7):e0181668. doi: 10.1371/journal.pone.0181668 (PMC5528883; doi:10.1371/journal.pone.0181668)

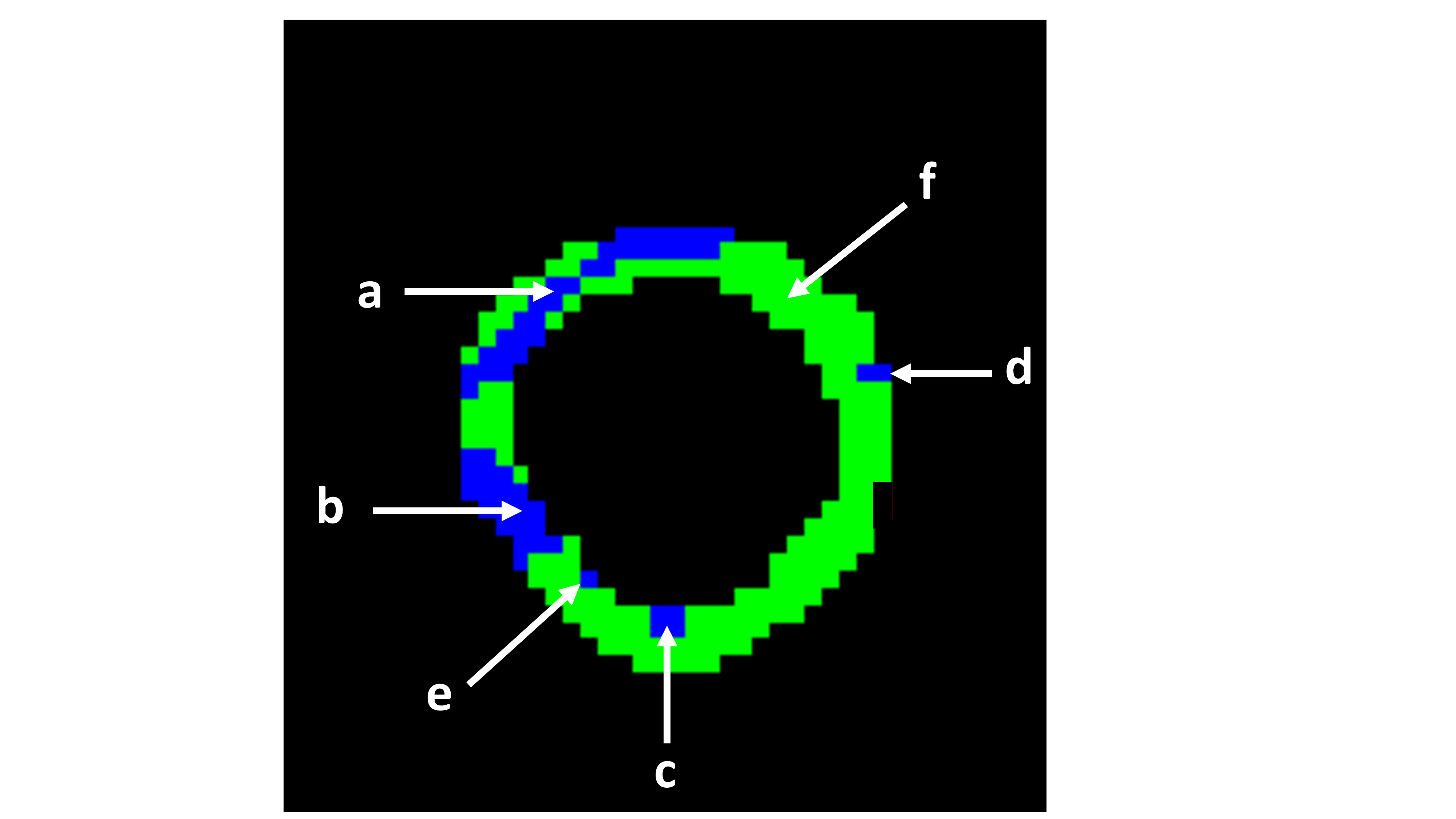

Supplement: S1 Fig — Cross-sectional slice of carotid artery demonstrating five different lipid deposits labelled alphabetically (a, b, c, d & e) and identified on T2 map technique. Lipid deposit (e) was excluded from the analysis as it counts ≤ 1%. LDD% is the percentage of deposit (a) out of the total lipid deposits (a/a + b + c + d) while RDD % = (1-a) or (b + c + d) divided by three in this example. (TIF) [file pone.0181668.s001.tif]
